# Overall survival at 5 years of follow-up in a phase III trial comparing ipilimumab 10 mg/kg with 3 mg/kg in patients with advanced melanoma

**DOI:** 10.1136/jitc-2019-000391

**Published:** 2020-06-04

**Authors:** Paolo Antonio Ascierto, Michele Del Vecchio, Andrzej Mackiewicz, Caroline Robert, Vanna Chiarion-Sileni, Ana Arance, Céleste Lebbé, Inge Marie Svane, Catriona McNeil, Piotr Rutkowski, Carmen Loquai, Laurent Mortier, Omid Hamid, Lars Bastholt, Brigitte Dreno, Dirk Schadendorf, Claus Garbe, Marta Nyakas, Jean-Jacques Grob, Luc Thomas, Gabriella Liszkay, Michael Smylie, Christoph Hoeller, Virginia Ferraresi, Florent Grange, Ralf Gutzmer, Joanna Pikiel, Fareeda Hosein, Burcin Simsek, Michele Maio

**Affiliations:** 1 Melanoma, Cancer Immunotherapy and Innovative Therapy Unit, Istituto Nazionale Tumori IRCCS Fondazione Pascale, Napoli, Italy; 2 Unit of Melanoma Medical Oncology, Fondazione IRCCS Istituto Nazionale dei Tumori, Milano, Lombardia, Italy; 3 Department of Diagnostics and Cancer Immunology, Greater Poland Cancer Center, Poznan Medical University, Poznan, Poland; 4 Department of Medicine, Dermatology Service, Gustave Roussy, Villejuif and Paris-Sud-University, Le Kremlin-Bicêtre, France; 5 Melanoma Oncology Unit, Istituto Oncologico Veneto-IRCCS, Padova, Italy; 6 Hospital Clinic and Institut D'Investigacions Biomèdiques August Pi i Sunyer, Barcelona, Spain; 7 Université de Paris, INSERM, Dermatology and CIC, Saint Louis Hospital, Paris, France; 8 Center for Cancer Immune Therapy, Herlev Hospital, Herlev, Denmark; 9 Department of Oncology, Copenhagen University Hospital, Herlev, Denmark; 10 Chris O’Brien Lifehouse and Royal Prince Alfred Hospital, Camperdown, New South Wales, Australia; 11 Department of Soft Tissue/Bone Sarcoma and Melanoma, Maria Skłodowska-Curie Institute—Oncology Center, Warsaw, Poland; 12 Department of Dermatology, University Medical Center, Mainz, Germany; 13 Clinique de Dermatologie, Unité d’Onco-Dermatologie, INSERM U1189, Centre Hospitalier Régional Universitaire de Lille, Hôpital Claude Huriez, Lille, France; 14 Melanoma Center, The Angeles Clinic and Research Institute, Los Angeles, California, USA; 15 Department of Oncology, Odense University Hospital, Odense, Denmark; 16 Department of Oncodermatology, University Hospital Centre Nantes, Nantes, Pays de la Loire, France; 17 Department of Dermatology, University Hospital Essen, Essen, Nordrhein-Westfalen, Germany; 18 Department of Dermatology, German Cancer Consortium, Heidelberg, Germany; 19 Department of Dermatology, Eberhard Karls Universitat Tübingen, Tübingen, Baden-Württemberg, Germany; 20 Department of Oncology, Oslo University Hospital, Oslo, Norway; 21 Dermatology and Skin Cancers Department, Aix-Marseille University, APHM, Marseille, France; 22 Department of Dermatology, Centre Hospitalier Lyon-Sud, Pierre-Bénite, France; 23 Department of Oncodermatology, National Institute of Oncology, Budapest, Hungary; 24 Department of Oncology, Cross Cancer Institute, Edmonton, Alberta, Canada; 25 Division of General Dermatology and Dermato-Oncology, Medical University of Vienna, Vienna, Austria; 26 Unit of Medical Oncology, IRCCS-Regina Elena National Cancer Institute, Rome, Italy; 27 Department of Dermatology, University Hospital Centre Reims, Reims, Champagne-Ardenne, France; 28 Operative Dermatology and Dermato-Oncology, Medizinische Hochschule Hannover, Hannover, Niedersachsen, Germany; 29 Department of Oncology, Wojewodzkie Centrum Oncologii, Gdańsk, Poland; 30 Oncology Clinical Development, Bristol-Myers Squibb Co, Princeton, New Jersey, USA; 31 Department of Biostatistics, Bristol-Myers Squibb Co, Princeton, New Jersey, USA; 32 Center for Immuno-Oncology, University Hospital of Siena, Instituto Toscano Tumori, Siena, Italy

**Keywords:** immunology, oncology, randomized trials

## Abstract

**Background:**

We have previously reported significantly longer overall survival (OS) with ipilimumab 10 mg/kg versus ipilimumab 3 mg/kg in patients with advanced melanoma, with higher incidences of adverse events (AEs) at 10 mg/kg. This follow-up analysis reports a 5-year update of OS and safety.

**Methods:**

This randomized, multicenter, double-blind, phase III trial included patients with untreated or previously treated unresectable stage III or IV melanoma. Patients were randomly assigned (1:1) to ipilimumab 10 mg/kg or 3 mg/kg every 3 weeks for 4 doses. The primary end point was OS.

**Results:**

At a minimum follow-up of 61 months, median OS was 15.7 months (95% CI 11.6 to 17.8) at 10 mg/kg and 11.5 months (95% CI 9.9 to 13.3) at 3 mg/kg (HR 0.84, 95% CI 0.71 to 0.99; p=0.04). In a subgroup analysis, median OS of patients with asymptomatic brain metastasis was 7.0 months (95% CI 4.0 to 12.8) in the 10 mg/kg group and 5.7 months (95% CI 4.2 to 7.0) in the 3 mg/kg group. In patients with wild-type or mutant *BRAF* tumors, median OS was 13.8 months (95% CI 10.2 to 17.0) and 33.2 months (95% CI 19.4 to 45.2) in the 10 mg/kg group, and 11.2 months (95% CI 9.2 to 13.8) and 19.7 months (95% CI 11.6 to 25.3) in the 3 mg/kg group, respectively. The incidence of grade 3/4 treatment-related AEs was 36% in the 10 mg/kg group vs 20% in the 3 mg/kg group, and deaths due to treatment-related AEs occurred in four (1%) and two patients (1%), respectively.

**Conclusions:**

This 61-month follow-up of a phase III trial showed sustained long-term survival in patients with advanced melanoma who started metastatic treatment with ipilimumab monotherapy, and confirmed the significant benefit for those who received ipilimumab 10 mg/kg vs 3 mg/kg. These results suggest the emergence of a plateau in the OS curve, consistent with previous ipilimumab studies.

**Trial registration number:**

NCT01515189.

## Introduction

Ipilimumab, an anticytotoxic T-lymphocyte antigen-4 (anti-CTLA-4) monoclonal antibody,^1^ was the first therapy to significantly improve overall survival (OS) in patients with advanced melanoma in a phase III trial.^2^ Since the approval of ipilimumab in 2011, the benchmark for survival in patients with advanced melanoma has been transformed.^2 3^ In a pooled ipilimumab analysis of patients with advanced melanoma, the survival curve plateaued at 3 years, with OS rates of approximately 20% sustained for up to 10 years.^4^ Ipilimumab 3 mg/kg is approved as both a first-line and a second-line therapy for the treatment of advanced melanoma in several countries. In addition, ipilimumab 10 mg/kg was approved as an adjuvant therapy in the USA, based on improved recurrence-free survival in patients with stage III melanoma,^5^ with an OS benefit demonstrated in a follow-up analysis.^6^

Because of the introduction of the antiprogrammed death-1 (anti-PD-1) agents nivolumab and pembrolizumab,^7–9^ ipilimumab is no longer commonly used as first-line monotherapy. Ipilimumab monotherapy is still an accepted treatment for some patients, such as those for whom anti-PD-1 treatment has failed. Ipilimumab 3 mg/kg in combination with nivolumab is used as a first-line therapy based on improved survival outcomes over monotherapy for patients with advanced melanoma.^7^

Previous studies have demonstrated a survival benefit with ipilimumab for patients with metastatic melanoma at the 10 mg/kg dose.^3 10^ Our initial phase III trial involving patients with advanced melanoma who had not received a prior BRAF or checkpoint inhibitor showed significantly longer OS with ipilimumab at 10 mg/kg than at 3 mg/kg, although with an increased incidence of adverse events (AEs).^11^ Here, we report a 5-year update of this trial, along with updated analyses of specific patient subgroups of clinical relevance.

## Patients and methods

### Patients

Details of the study design and eligibility criteria have been described previously.^11^ Eligible patients were aged ≥18 years and had untreated or previously treated unresectable stage III/IV metastatic melanoma, an Eastern Cooperative Oncology Group performance status (ECOG PS) score of 0 or 1 and measurable disease within 28 days of the first dose of study treatment, based on modified WHO diagnostic criteria. Patients who had received prior therapy with BRAF inhibitors, CTLA-4 or PD-1 antagonists or programmed death-ligand 1 or CD137 agonists were excluded, as were patients with symptomatic brain metastases or brain metastases requiring treatment, a history of autoimmune disease or a diagnosis of primary ocular melanoma.

### Study design

This randomized, multicenter, double-blind, phase III study was conducted at 87 centers in 21 countries, with the majority of patients enrolled in Europe. Patients were randomly assigned 1:1 to receive ipilimumab 10 mg/kg or 3 mg/kg, and were stratified by metastatic (M) substage (M0/M1a/M1b or M1c without brain metastases or M1c with brain metastases), previous treatment for metastatic melanoma (yes or no) and an ECOG PS of 0 or 1. The randomization and masking methods have been described previously.^11^

Ipilimumab was administered by intravenous infusion for 90 min every 3 weeks for four doses (without the opportunity for maintenance therapy) until disease progression per immune-related response criteria,^12 13^ unacceptable toxicity or withdrawal of consent (initial treatment phase). In addition, patients with a complete or partial response or stable disease for ≥3 months and subsequent progression were eligible for re-treatment with ≤4 doses of originally assigned ipilimumab treatment (re-treatment phase). Tumor response was assessed by investigators at weeks 12, 16 and 24, and then every 12 weeks. Discontinuation criteria were based on immune-related response criteria to account for the unconventional response patterns observed with ipilimumab (responses that occur after an initial increase in tumor volume or the observation of new lesions).^12 13^ Dose reduction was not permitted; however, dosing was delayed for all-cause skin-related AEs grade ≥3 and could be delayed for treatment-related AEs and laboratory abnormalities, per the investigator.

### End points and assessments

The primary end point was OS. Secondary end points included the yearly assessment of OS for up to 5 years, OS based on brain metastases, objective response, progression-free survival and safety. Descriptive analyses of OS in several patient subgroups, as well as updated safety in patients who had received ≥1 dose of study treatment for ≤90 days after the last dose of study drug, are also presented. As opposed to the initial report, which included an analysis of safety in the initial treatment phase only, the current update includes AEs collected during the re-treatment phase. AE severity was graded based on National Cancer Institute Common Terminology Criteria for Adverse Events, V.3.0.^14^

### Statistical analysis

Sample size determination has been described previously.^11^ A stratified log-rank test was used to compare OS among the randomized groups. HRs and associated two-sided 95% CIs were estimated using a stratified Cox model, with the randomized group being the only covariate. Event-free OS probabilities were estimated using the Kaplan-Meier method. Estimates of medians and corresponding 95% CIs were calculated using the Brookmeyer and Crowley method.^15^ Statistical analyses were performed using SAS V.9.3 and V.9.4. OS subgroup analyses were not powered to evaluate statistical significance.

## Results

### Patients

Patient disposition and baseline characteristics were described previously.^11^ To summarize, 831 patients were enrolled between February 29, 2012 and July 9, 2012, among whom 727 patients were randomized 1:1 to receive ipilimumab 10 mg/kg (n=365) or 3 mg/kg (n=362); 23 and 32 patients received first re-treatment, respectively, and 2 in each group received second re-treatment (online supplementary figure S1). One patient in the ipilimumab 10 mg/kg group experienced an AE, was not treated and was excluded from the safety population. As reported previously, baseline characteristics were comparable in the two treatment groups as a whole (online supplementary table S1) and among the 22% of patients in each group with *BRAF* mutation-positive tumors.^11^ At database lock (September 13, 2017), patients had received a median (range) of 4(1–16) and 4(1–11) doses of ipilimumab in the 10 mg/kg and 3 mg/kg groups, respectively. Subsequent systemic therapy was received by 38% and 39% of patients in the 10 mg/kg and 3 mg/kg groups, respectively, including immunotherapy in 18% and 15% of patients and targeted therapy in 10% and 13% of patients (online supplementary table S2).

10.1136/jitc-2019-000391.supp1 Supplementary data

### Efficacy

**Figure 1:**
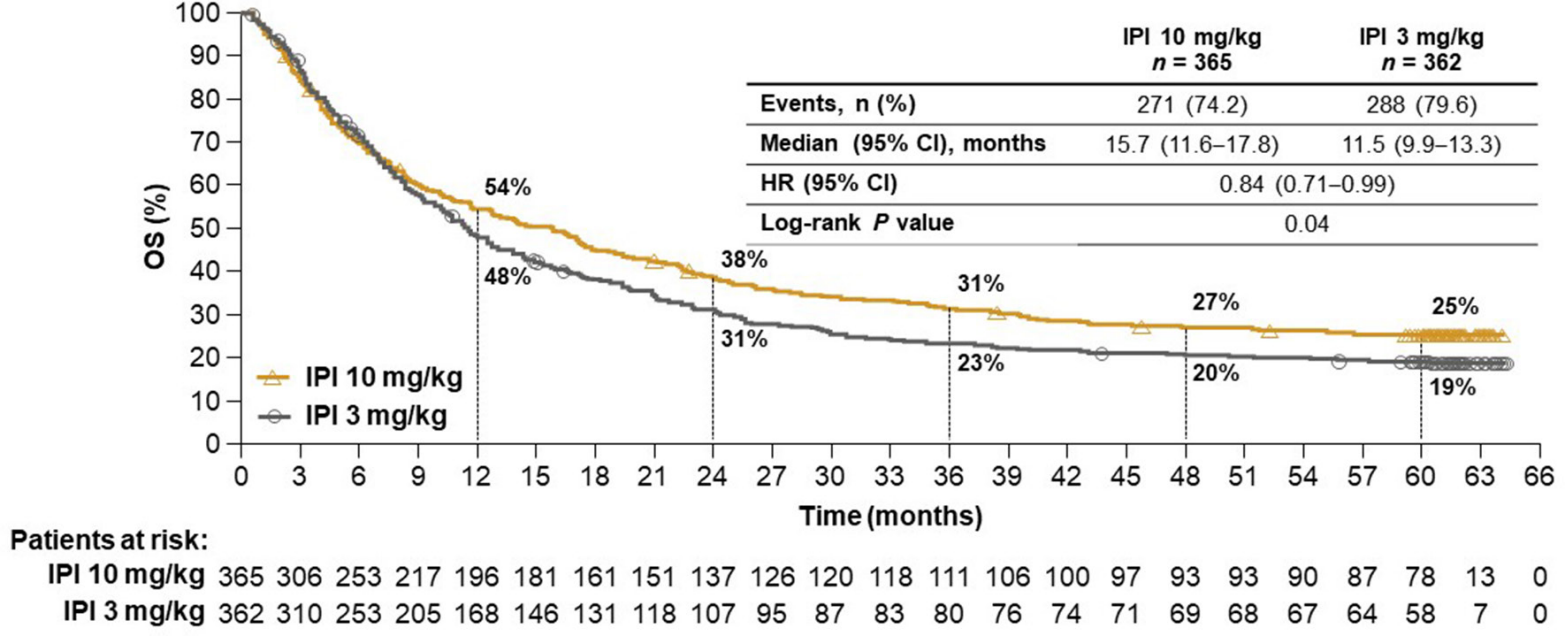
Overall survival in all randomized patients. IPI, ipilimumab.

At database lock, patients had been followed for a minimum of 61 months, with a median follow-up of 14.5 months (range 0.6‒64.0) and 11.2 months (range 0.1‒64.2) in the 10 mg/kg and 3 mg/kg groups, respectively. Consistent with the initial analysis,^11^ OS was significantly longer in the 10 mg/kg group compared with the 3 mg/kg group (HR 0.84, 95% CI 0.71 to 0.99; p=0.04), with a median OS of 15.7 months (95% CI 11.6 to 17.8) and 11.5 months (95% CI 9.9 to 13.3), respectively (figure 1). Five-year survival rates were 25% (95% CI 21 to 29) and 19% (95% CI 15 to 23) in the 10 mg/kg and 3 mg/kg groups, respectively.

**Figure 2:**
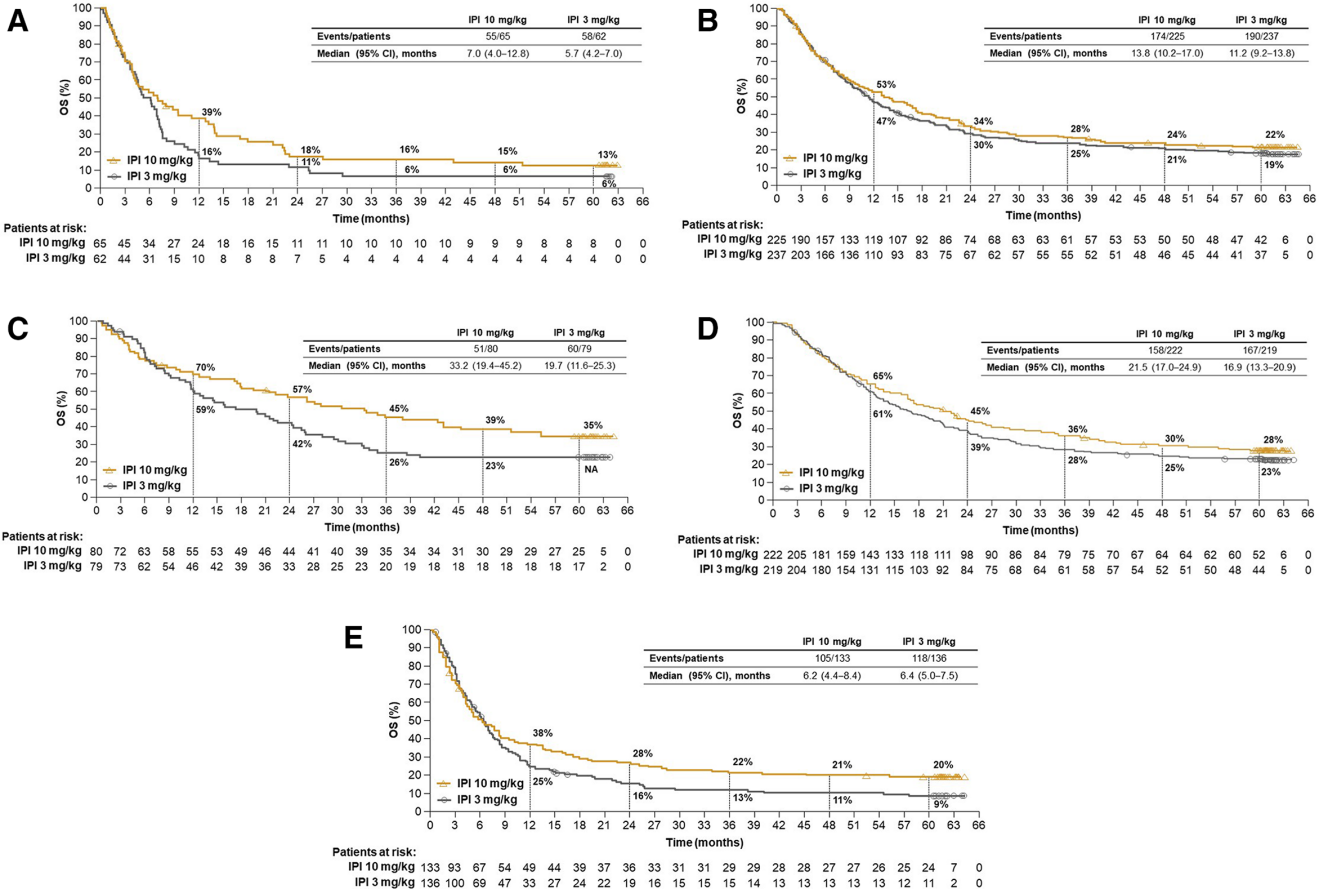
Overall survival by subgroups. (A) Overall survival in patients with asymptomatic brain metastases at baseline, (B) wild-type *BRAF* tumors, (C) mutant *BRAF* tumors, (D) LDH levels ≤ULN and (E) LDH levels >ULN. IPI, ipilimumab; LDH, lactate dehydrogenase; NA, not available; OS, overall survival; ULN, upper limit of normal.

**Figure 3:**
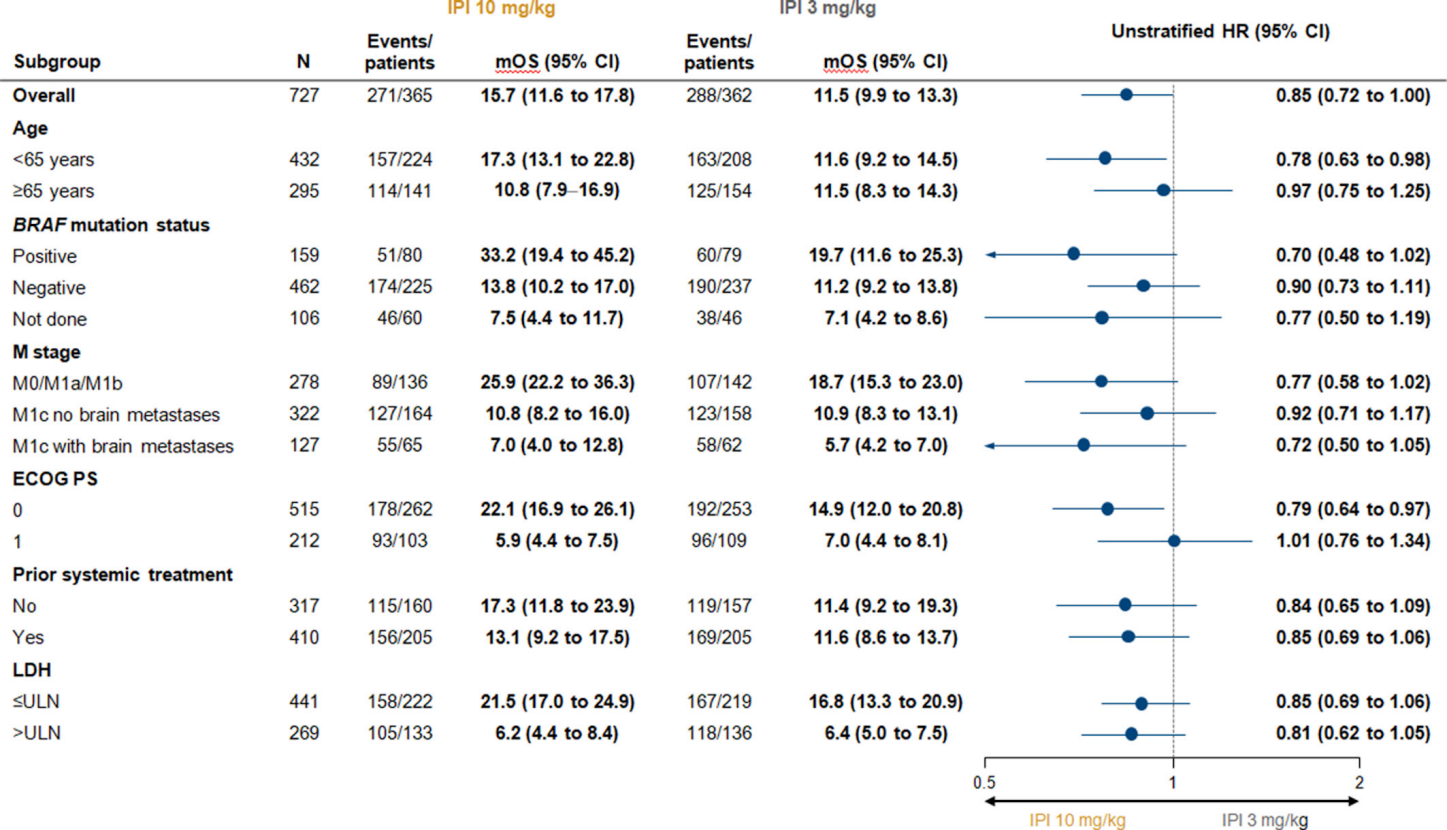
Forest plot of overall survival. ECOG PS, Eastern Cooperative Oncology Group performance status, IPI, ipilimumab; LDH, lactate dehydrogenase; M, metastatic; mOS, median overall survival; ULN, upper limit of normal.

Descriptive OS analyses were also performed in several patient subgroups of clinical relevance. Among patients with asymptomatic brain metastasis at baseline, median OS was 7.0 months (95% CI 4.0 to 12.8) in the 10 mg/kg group and 5.7 months (95% CI 4.2 to 7.0) in the 3 mg/kg group, with 5-year OS rates of 13.0% (95% CI 6 to 23) and 6% (95% CI 2 to 14), respectively (figure 2A). In patients with wild-type *BRAF* tumors treated with the 10 mg/kg and 3 mg/kg doses, median OS was 13.8 months (95% CI 10.2 to 17.0) and 11.2 months (95% CI 9.2 to 13.8), respectively, with 5-year survival rates of 22% (95% CI 17 to 28) and 19% (95% CI 14 to 24) (figure 2B). In patients with mutant *BRAF* tumors, median OS was 33.2 months (95% CI 19.4 to 45.2) and 19.7 months (95% CI 11.6 to 25.3) in the 10 mg/kg and 3 mg/kg groups, respectively. The 5-year OS rate was 35% (95% CI 25 to 46) in the 10 mg/kg group (figure 2C), but could not be calculated for the 3 mg/kg group because of missing patient data (the 4-year rate for the 3 mg/kg group was 23% [95% CI 15 to 33]). Five-year OS rates were 28% (95% CI 22 to 34) and 23% (95% CI 18 to 29) in patients with lactate dehydrogenase (LDH) levels less than or equal to the upper limit of normal (ULN) treated with the 10 mg/kg and 3 mg/kg doses, respectively (figure 2D), and 20% (95% CI 14 to 27) and 9% (95% CI 5 to 15) in patients with LDH levels greater than the ULN treated with the 10 mg/kg and 3 mg/kg doses, respectively (figure 2E). OS in other subgroups also showed trends favoring the 10 mg/kg dose (figure 3).

### Safety

Given that safety updates from the previous analysis would have been from re-treatment only, results were very consistent.^11^ A greater proportion of patients in the ipilimumab 10 mg/kg group experienced treatment-related AEs (any grade, 79%; grade 3/4, 36%) compared with the 3 mg/kg group (any grade, 64%; grade 3/4, 20%). The most common grade 3/4 treatment-related AEs were diarrhea (11%), colitis (6%) and increased alanine aminotransferase (4%) for patients in the 10 mg/kg group, and diarrhea (6%), colitis (3%) and hypophysitis (2%) for those in the 3 mg/kg group. In total, 34% and 19% of patients discontinued treatment because of AEs from any cause in the 10 mg/kg and 3 mg/kg groups, respectively, including 26% and 12% of patients because of grade 3/4 AEs (table 1). The most frequently reported AEs leading to discontinuation in both groups were diarrhea, at 8% and 4%, and colitis, at 4% and 2%, respectively. Immune-related AEs (those identified by the investigator as treatment related and associated with an immune-mediated mechanism) were observed in 74% and 55% of patients in the 10 mg/kg and 3 mg/kg groups, respectively (online supplementary table S3); the most common in both groups were diarrhea (39% and 23%), rash (26% and 15%) and pruritus (23% and 23%). Re-treatment for progressive disease with either dose did not result in increased toxicity.

**Table 1:** Adverse events

	Ipilimumab 10 mg/kg (n=364)				Ipilimumab 3 mg/kg (n=362)			
	Any grade	Grade 3	Grade 4	Grade 5	Any grade	Grade 3	Grade 4	Grade 5
AEs of any cause	347 (95)	129 (35)	41 (11)	58 (16)	341 (94)	105 (29)	33 (9)	58 (16)
AEs of any cause leading to discontinuation	122 (34)	72 (20)	21 (6)	8 (2.2)	70 (19)	33 (9)	12 (3)	14 (4)
Treatment-related AEs*	288 (79)	105 (29)	26 (7)	1 (<1)	233 (64)	61 (17)	10 (3)	0
Diarrhea	142 (39)	38 (10)	1 (<1)	0	85 (23)	21 (6)	0	0
Rash	95 (26)	6 (2)	0	0	53 (15)	2 (1)	0	0
Pruritus	83 (23)	3 (1)	0	0	85 (23)	2 (1)	0	0
Fatigue	41 (11)	3 (1)	0	0	36 (10)	4 (1)	0	0
Colitis	39 (11)	20 (5)	2 (1)	0	20 (6)	9 (2)	1 (<1)	0
Asthenia	32 (9)	5 (1)	0	0	20 (6)	1 (<1)	0	0
Increased alanine aminotransferase	29 (8)	11 (3)	3 (1)	0	5 (1)	1 (<1)	1 (<1)	0
Increased aspartate aminotransferase	25 (7)	6 (2)	2 (1)	0	4 (1)	1 (<1)	0	0
Hypophysitis	24 (7)	9 (2)	1 (<1)	0	14 (4)	6 (2)	3 (1)	0
Fever	23 (6)	1 (<1)	0	0	18 (5)	0	0	0
Nausea	21 (6)	1 (<1)	0	0	27 (7)	0	0	0
Headache	21 (6)	3 (1)	1 (<1)	0	18 (5)	1 (<1)	0	0
Multifocal colon perforation	0	0	0	1 (<1)	0	0	0	0

All data are n (%).

*Any-grade treatment-related AEs occurring in ≥5% of patients and all grade 5 events are shown.

AE, adverse event.

In the ipilimumab 10 mg/kg and 3 mg/kg groups, respectively, 74% and 80% of patients died, with the primary cause of death being progressive disease for most of these patients (68% and 73%). As previously reported, deaths as the result of treatment-related toxicity occurred in four patients in the 10 mg/kg group (diarrhea leading to general deterioration, fulminant colitis, multiorgan failure and bowel perforation) and two patients in the 3 mg/kg group (multifocal colon perforation and myocardial infarction from complications of diarrhea and colitis).^11^ No treatment-related death was reported following the initial analysis.

## Discussion

Updated results of this phase III trial in patients with advanced melanoma who had not received a prior BRAF or checkpoint inhibitor demonstrated a significant improvement in OS with ipilimumab monotherapy at 10 mg/kg vs 3 mg/kg (four doses during induction or re-treatment, without maintenance therapy). This benefit was sustained after 61 months of follow-up. These results suggest the emergence of a survival plateau that was sustained at 5 years. Similar results were previously observed in a pooled analysis of ipilimumab studies that reported an OS rate of 19% at 5 years, with a plateau starting at 3 years.^4^ Consistent with the original analysis,^11^ ipilimumab 10 mg/kg was associated with higher incidences of treatment-related AEs and AEs leading to discontinuation than ipilimumab 3 mg/kg.

Although the treatment paradigm for metastatic melanoma has shifted with the use of anti-PD-1 checkpoint inhibitors alone or in combination with ipilimumab, ipilimumab monotherapy may still be a consideration, such as in the treatment of particular patient subgroups following failure of anti-PD-1 therapy.^16^ In this study, in patients with wild-type *BRAF* tumors, long-term survival with ipilimumab at either dose was similar to that of the overall population, and patients with *BRAF* mutations also benefitted from ipilimumab therapy. Moreover, the subgroup of patients with *BRAF* mutations had improved OS compared with those with wild-type *BRAF*, an observation that was especially evident in the 10 mg/kg group, in which median OS was 33.2 months (vs 13.8 months for patients with wild-type *BRAF*). Of note, the numbers of patients in the two groups differed greatly, with 80 patients having a *BRAF* mutation compared with 225 patients with wild-type *BRAF*. Patients with asymptomatic brain metastasis also showed long-term benefit with ipilimumab. However, OS in these subgroups may have been affected by low patient numbers.

The overall safety profile of long-term treatment with ipilimumab 10 mg/kg or 3 mg/kg was manageable and no new safety concerns were identified. Because few patients continued to receive treatment after the initial analysis, the updated safety results were similar to those reported previously, with greater toxicity with the higher dose. Previous results also showed that most AEs resolved using established management algorithms, and similar resolution of AEs between the two dose groups was observed.^11^

Results presented here add to those available on whether ipilimumab effects may be dose-dependent. Previously, a retrospective analysis of 498 patients showed that higher doses of ipilimumab were associated with steady-state trough concentrations that may have in turn been associated with increased tumor responses, longer survival and higher rates of immune-related AEs.^17^ In addition, previous reports have shown a dose-dependent effect of ipilimumab on response^10 17^; however, effects on survival was not shown. A recent phase II study in patients who received at least one previous treatment reported a non-statistical dose effect on progression-free survival (PFS), but not OS.^18^ In contrast, results from the study presented here showed a significant improvement in OS, but not PFS, with 10 mg/kg vs 3 mg/kg.^11^ Taking these results together, further investigation is needed to answer the important question of potential ipilimumab efficacy and dose-dependency. Of note, ipilimumab-related toxicity has been shown consistently to be dose-dependent in melanoma.^10 11 17 19^

There were a few notable limitations to this study. The enrollment criteria, which were established based on the treatment landscape at the time of the study design, excluded patients who had received prior therapy, precluding analysis of ipilimumab as second-line treatment. The survival results may have been confounded by therapy received after ipilimumab. However, in the previous report, post hoc analyses by subsequent systemic therapy showed the consistent benefit of the 10 mg/kg dose over the 3 mg/kg dose.^11^ It should be additionally noted that the study reflects the dose effect of anti-CTLA-4 at a time when few patients could receive anti-PD-1 as a second-line therapy. Although OS across subgroups generally favored ipilimumab 10 mg/kg, the study was not powered for subgroup analyses. Finally, the previous study showed more reductions in quality of life (QoL) scales at the higher dose in the initial treatment phase, which may have reflected greater toxicity.^11^ However, with many patients alive years after coming off study treatments, coupled with the use of poststudy treatment with other agents, in many cases, long-term QoL evaluation provides little information about ipilimumab treatment.

The results of this study may be useful in offering insights into the development of new anti-CTLA-4 agents. CTLA-4-NF (NCT03110107) and CTLA-4-Probody (NCT03369223) are two ipilimumab-based compounds that are being tested in patients with solid tumors, including melanoma. Other novel anti-CTLA-4 agents in early stage clinical trials in solid tumors, including melanoma, are AGEN1884, ADU-1604 and MK1308.

In this follow-up analysis of patients with advanced melanoma who were initially treated with ipilimumab monotherapy, the superiority of the survival benefit of the 10 mg/kg vs the 3 mg/kg dose was sustained over the long term, and this survival benefit was observed in clinically relevant subgroups. However, the higher dose was associated with greater toxicity, although no new safety concerns were identified. Consequently, initial ipilimumab monotherapy dosing appeared to be relevant to outcomes. These data may have implications for the evaluation and treatment sequencing of investigational anti-CTLA-4 agents.
