# Correction: *Overall survival at 5 years of follow-up in a phase III trial comparing ipilimumab 10 mg/kg with 3 mg/kg in patients with advanced melanoma*

**DOI:** 10.1136/jitc-2019-000391corr1

**Published:** 2020-07-12

Ascierto PA, Del Vecchio M, Mackiewicz A, *et al.* Overall survival at 5 years of follow-up in a phase III trial comparing ipilimumab 10 mg/kg with 3 mg/kg in patients with advanced melanoma. *J ImmunoTher Cancer* 2020;8:e000391. doi: 10.1136/jitc-2019-000391

Since the online publication of this article, it was noticed that ‘Michele Del Vecchio’ was incorrectly spelt as ‘Michelle Del Vecchio’. This error has been corrected.

