# Supplementary material for: Overall survival at 5 years of follow-up in a phase III trial comparing ipilimumab 10 mg/kg with 3 mg/kg in patients with advanced melanoma
Source: J Immunother Cancer. 2020 Jun 4;8(1):e000391. doi: 10.1136/jitc-2019-000391 (PMC7279645; doi:10.1136/jitc-2019-000391)
Supplement: Supplementary data [file jitc-2019-000391supp001.pdf]

Supplementary Appendix

Supplement to: Ascierto PA, Del Vecchio M, Robert C, Chiarion-Sileni V, Arance A, Lebbé C, et al. Overall survival at 5 years of follow-up in a phase 3 trial comparing ipilimumab 10 mg/kg with 3 mg/kg in patients with advanced melanoma

Ascierto PA, *et al. J Immunother Cancer* 2020; 8:e000391. doi: 10.1136/jitc-2019-000391

Fig. S1. Trial design. Abbreviations: *ITT* intention-to-treat, *OS* overall survival.

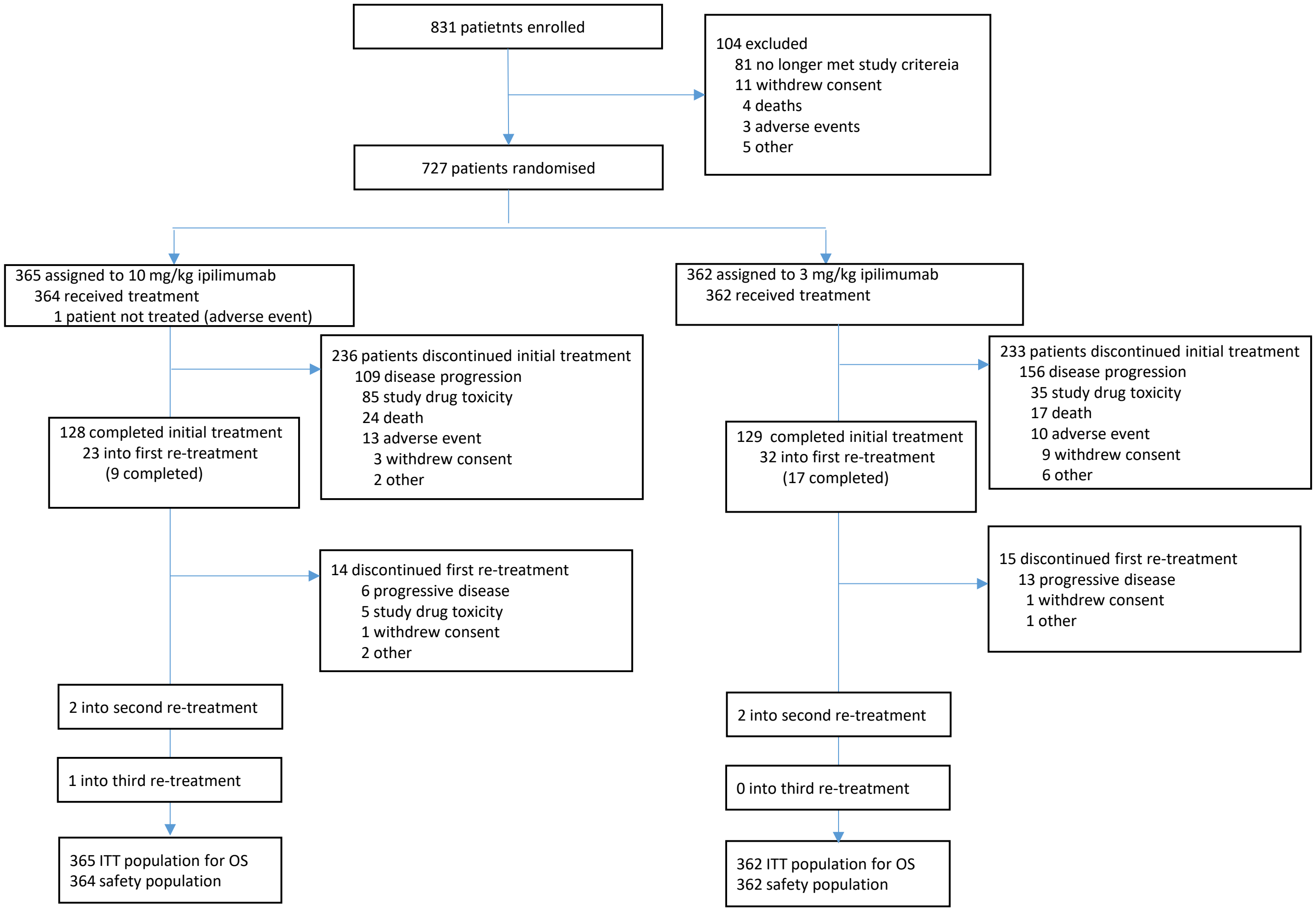

Ascierto PA, *et al. J Immunother Cancer* 2020; 8:e000391. doi: 10.1136/jitc-2019-000391

Table S1. Baseline patient and disease characteristics (11)

**Characteristic**	**Ipilimumab 10 mg/kg (*n* = 365)**	**Ipilimumab 3 mg/kg (*n* = 362)**
Age (years)		
Median (IQR)	62 (49–70)	62 (51–71)
Age, *n* (%)		
< 65 years	224 (61)	208 (57)
≥ 65 years	141 (39)	154 (43)
Sex, *n* (%)		
Male	219 (60)	231 (64)
Female	146 (40)	131 (36)
ECOG PS, *n* (%)		
0	262 (72)	253 (70)
1	103 (28)	109 (30)
M stage, *n* (%)		
M0	19 (5)	20 (6)
M1a	45 (12)	45 (12)
M1b	72 (20)	77 (21)
M1c without brain metastases	164 (45)	158 (44)
M1c with brain metastases	65 (18)	62 (17)
Prior treatment for melanoma, *n* (%)		
Yes	205 (56)	205 (57)
No	160 (44)	157 (43)
Prior therapy, *n* (%)		
Any prior systemic therapy[a]	206 (56)	205 (57)
Any prior radiotherapy	107 (29)	96 (27)
Any prior surgery	359 (98)	346 (96)
Lactate dehydrogenase, *n* (%)		
≤ ULN	222 (61)	219 (60)
> ULN	133 (36)	136 (38)
≤ 2 × ULN	321 (88)	306 (85)
> 2 × ULN	34 (9)	49 (14)
Not reported	10 (3)	7 (2)
AJCC disease stage, *n* (%)		
III	35 (10)	35 (10)
IV	330 (90)	327 (90)
BRAF status, *n* (%)		
V600	75 (21)	75 (21)
Other mutation	5 (1)	4 (1)
No mutation	225 (62)	237 (65)
Unknown	60 (16)	46 (13)

Ascierto PA, *et al. J Immunother Cancer* 2020; 8:e000391. doi: 10.1136/jitc-2019-000391

[a]No patients received previous treatment with a BRAF inhibitor.

Abbreviations: *AJCC* American Joint Committee on Cancer, *ECOG PS* Eastern Cooperative Oncology Group performance status, *IQR* interquartile range, *M* metastatic, *ULN* upper limit of normal.

Ascierto PA, *et al. J Immunother Cancer* 2020; 8:e000391. doi: 10.1136/jitc-2019-000391

Table S2. Subsequent therapy received by randomized patients off study[a]

	**Ipilimumab 10 mg/kg (*n* = 365)**	**Ipilimumab 3 mg/kg (*n* = 362)**
**Any subsequent systemic therapy**	137 (38)	141 (39)
Chemotherapy	104 (28)	111 (31)
Any immunotherapy	64 (18)	55 (15)
Ipilimumab	13 (4)	10 (3)
Anti–PD-1 agent	47 (13)	40 (11)
Interferon/interferon-alpha	4 (1)	1 (<1)
Interleukin/interleukin-2	2 (1)	4 (1)
Investigational immunotherapy	11 (3)	9 (2)
T-cell infusion	0	2 (1)
BRAF/MEK inhibitor	37 (10)	48 (13)
Other	2 (1)	2 (1)
**Radiotherapy**	100 (27)	115 (32)
**Surgery**	82 (22)	82 (23)

All data are n (%).

[a]Patients may have received more than one subsequent systemic therapy.

Abbreviation: *PD-1* programmed death-1.

Ascierto PA, *et al. J Immunother Cancer* 2020; 8:e000391. doi: 10.1136/jitc-2019-000391

Table S3. Immune-related AEs[a]

	Ipilimumab 10 mg/kg (*n* = 364)			Ipilimumab 3 mg/kg (*n* = 362)		
	**Any grade**	**Grade 3**	**Grade 4**	**Any grade**	**Grade 3**	**Grade 4**
**Any immune-related AE**	271 (74)	97 (27)	20 (5)	200 (55)	47 (13)	8 (2)
**Skin and subcutaneous**	163 (45)	8 (2)	0	137 (38)	5 (1)	0
Rash	95 (26)	6 (2)	0	53 (15)	2 (1)	0
Pruritus	83 (23)	3 (1)	0	85 (23)	2 (1)	0
Maculopapular rash	5 (1)	1 (<1)	0	4 (1)	0	0
Pruritic rash	5 (1)	0	0	4 (1)	1 (<1)	0
Toxic skin eruption	1 (<1)	0	0	1 (<1)	1 (<1)	0
**Gastrointestinal**	148 (41)	51 (14)	6 (2)	92 (25)	28 (8)	4 (1)
Diarrhea	142 (39)	38 (10)	1 (<1)	85 (23)	21 (6)	0
Colitis	39 (11)	20 (5)	2 (1)	20 (6)	9 (2)	1 (<1)
Autoimmune colitis	4 (1)	3 (1)	0	5 (1)	3 (1)	1 (<1)
Ulcerative colitis	3 (1)	2 (1)	0	1 (<1)	0	1 (<1)
Intestinal perforation	2 (1)	0	2 (1)	0	0	0
Gastrointestinal perforation	0	0	0	1 (<1)	0	1 (<1)
Autoimmune pancreatitis	1 (<1)	0	1 (<1)	0	0	0
Large intestine haemorrhage	1 (<1)	1 (<1)	0	0	0	0
Large intestine perforation	1 (<1)	1 (<1)	0	1 (<1)[b]	0	0
Rectal haemorrhage	1 (<1)	0	0	2 (1)	1 (<1)	0
Small intestine perforation	0	0	0	1 (<1)	0	1 (<1)
**Endocrine**	52 (14)	18 (5)	1 (<1)	37 (10)	8 (2)	3 (1)
Hypophysitis	24 (7)	9 (2)	1 (<1)	14 (4)	6 (2)	3 (1)
Hypopituitarism	8 (2)	4 (1)	0	5 (1)	2 (1)	0
Hypothyroidism	8 (2)	1 (<1)	0	7 (2)	0	0
Thyroiditis	5 (1)	1 (<1)	0	2 (1)	0	0
Adrenal insufficiency	3 (1)	2 (1)	0	5 (1)	0	0
Adrenocorticotropic hormone deficiency	2 (1)	1 (<1)	0	2 (1)	0	0
Acute adrenocortical insufficiency	1 (<1)	1 (<1)	0	1 (<1)	0	1 (<1)
Lymphocytic hypophysitis	1 (<1)	1 (<1)	0	2 (1)	1 (<1)	0

	**Ipilimumab 10 mg/kg (*n* = 364)**			**Ipilimumab 3 mg/kg (*n* = 362)**		
	**Any grade**	**Grade 3**	**Grade 4**	**Any grade**	**Grade 3**	**Grade 4**
Thyrotoxic crisis	0	0	0	1 (<1)	0	1 (<1)
**Investigations**	44 (12)	17 (5)	6 (2)	9 (2)	2 (1)	1 (<1)
Increased alanine aminotransferase	29 (8)	11 (3)	3 (1)	5 (1)	1 (<1)	1 (<1)
Increased aspartate aminotransferase	25 (7)	6 (2)	2 (1)	4 (1)	1 (<1)	0
Increased transaminases	5 (1)	2 (1)	1 (<1)	3 (1)	1 (<1)	0
Increased gamma-glutamyltransferase	4 (1)	2 (1)	0	3 (1)	1 (<1)	1 (<1)
Increased hepatic enzyme	3 (1)	1 (<1)	0	0	0	0
Increased liver function test	2 (1)	1 (<1)	1 (<1)	0	0	0
Abnormal alanine aminotransferase	1 (<1)	1 (<1)	0	0	0	0
Increased amylase	1 (<1)	1 (<1)	0	0	0	0
**Liver**	22 (6)	11 (3)	8 (2)	7 (2)	6 (2)	0
Hepatocellular injury	6 (2)	3 (1)	2 (1)	1 (<1)	0	0
Hepatitis	5 (1)	4 (1)	1 (<1)	2 (1)	2 (1)	0
Autoimmune hepatitis	4 (1)	2 (1)	2 (1)	2 (1)	1 (<1)	0
Hepatotoxicity	3 (1)	2 (1)	1 (<1)	2 (1)	2 (1)	0
Acute hepatic failure	1 (<1)	0	1 (<1)	0	0	0
Acute hepatitis	1 (<1)	0	1 (<1)	0	0	0
Hyperbilirubinemia	0	0	0	1 (<1)	1 (<1)	0
**Immune system disorders**	14 (4)	3 (1)	0	1 (<1)	0	0
Hypersensitivity	13 (4)	3 (1)	0	1 (<1)	0	0
**Nervous system disorders**	11 (3)	4 (1)	0	3 (1)	1 (<1)	0
Guillain-Barré syndrome	2 (1)	2 (1)	0	0	0	0
Peripheral sensory neuropathy	2 (1)	2 (1)	0	0	0	0
Peripheral motor neuropathy	1 (<1)	1 (<1)	0	0	0	0
Neuralgia	0	0	0	1 (<1)	1 (<1)	0
**Respiratory, thoracic and mediastinal disorders**	4 (1)	3 (1)	1 (<1)	0	0	0
Pneumonitis	4 (1)	3 (1)	1 (<1)	0	0	0

	Ipilimumab 10 mg/kg (*n* = 364)			Ipilimumab 3 mg/kg (*n* = 362)		
	Any grade	Grade 3	Grade 4	Any grade	Grade 3	Grade 4
**Metabolism and nutritional disorders**	2 (1)	1 (<1)	1 (<1)	0	0	0
Hyperglycemia	1 (<1)	1 (<1)	0	0	0	0
Hyperlipasemia	1 (<1)	0	1 (<1)	0	0	0
**General disorders**	1 (<1)	1 (<1)	0	0	0	0
Multi-organ dysfunction	1 (<1)	1 (<1)	0	0	0	0
**Infections and infestations**	2 (1)	0	0	3 (1)	0	1 (<1)
Peritonitis	0	0	0	1 (<1)	0	1 (<1)
**Eye disorders**	0	0	0	2 (1)	1 (<1)	0
Uveitis	0	0	0	2 (1)	1 (<1)	0

All data are *n* (%). AEs with any reported grade 3 or higher event are included.

[a]Immune-related AEs are both AEs of interest and those considered treatment-related by the investigator. AEs of interest are those consistent with an immune-mediated mechanism and include enterocolitis, dermatitis, hepatitis, endocrinopathies, and neuropathies; however, other less common immune-mediated AEs have also been reported. The severity of these immune-related AEs may range from mild to severe and life-threatening.

[b]Grade 5 event.

Abbreviation: AE, adverse event.

Ascierto PA, *et al. J Immunother Cancer* 2020; 8:e000391. doi: 10.1136/jitc-2019-000391
